# Supplementary material for: Association between emergency department attendances, sociodemographic factors and long-term health conditions in the population of Norfolk and Waveney, England: Cross sectional study
Source: PLoS One. 2024 May 8;19(5):e0303270. doi: 10.1371/journal.pone.0303270 (PMC11078347; doi:10.1371/journal.pone.0303270)
Supplement: S1 File — (DOCX) [file pone.0303270.s001.docx]

**Supporting information**

**Supplementary Table S1. Association between explanatory variables and frequency of emergency department visits in total population: Poison regression mixed models with general practice as random effect**

| Variable | IRR | 95% CI | | p-value |
| --- | --- | --- | --- | --- |
| Age <=4 | 1.2576 | 1.2363 | 1.2793 | <0.001 |
| Age 5-14 | 0.9130 | 0.8993 | 0.9269 | <0.001 |
| Age 15-35 (ref) | -- | -- | -- | -- |
| Age 36-70 | 0.7189 | 0.7111 | 0.7268 | <0.001 |
| Age >70 | 0.9220 | 0.9112 | 0.9328 | <0.001 |
| Female (ref) | -- | -- | -- | -- |
| Male | 1.0151 | 1.0070 | 1.0233 | <0.001 |
| White (ref) | -- | -- | -- | -- |
| Not known/missing | 0.1138 | 0.1120 | 0.1157 | <0.001 |
| Other | 1.0012 | 0.9794 | 1.0235 | 0.92 |
| IMD (continuous per decile) | 0.9671 | 0.9652 | 0.9690 | <0.001 |
| Distance (10km) | 0.8592 | 0.8398 | 0.8792 | <0.001 |
| Distance square (100km^2^) | 1.0233 | 1.0183 | 1.0283 | <0.001 |
| Effect of GP surgery (random effect) | Variance  0.02131 | SD  0.146 | No. GPs  91 |  |
| IRR Incidence rate ratio, CI confidence interval, IMD Index of Multiple Deprivation. GP general practice | | | | |

**Supplementary Table S2. Association between explanatory variables and frequency of emergency department visits in individuals with long term condition and general practice appointment data: Poison regression mixed models with general practice as random effect**

|  | Model 1 | | | | Model 2 | | | | Model 3 | | | | Model 4 | | | |
| --- | --- | --- | --- | --- | --- | --- | --- | --- | --- | --- | --- | --- | --- | --- | --- | --- |
| Variable | IRR | 95% CI | | p-value | IRR | 95% CI | | p-value | IRR | 95% CI | | p-value | IRR | 95% CI | | p-value |
| Age <=4 | 1.9535 | 1.8527 | 2.0597 | <0.001 | 2.1187 | 2.0094 | 2.2340 | <0.001 | 2.0745 | 1.9667 | 2.1882 | <0.001 | 1.8926 | 1.7919 | 1.9990 | <0.001 |
| Age 5-14 | 0.9568 | 0.9297 | 0.9847 | 0.003 | 1.0167 | 0.9879 | 1.0464 | 0.26 | 1.0617 | 1.0311 | 1.0932 | <0.001 | 1.1560 | 1.1203 | 1.1928 | <0.001 |
| Age 15-35 (ref) | -- | -- | -- | -- | -- | -- | -- | -- | -- | -- | -- | -- | -- | -- | -- | -- |
| Age 36-70 | 0.6295 | 0.6208 | 0.6384 | <0.001 | 0.5331 | 0.5255 | 0.5407 | <0.001 | 0.5992 | 0.5905 | 0.6079 | <0.001 | 0.5943 | 0.5852 | 0.6034 | <0.001 |
| Age >70 | 0.7602 | 0.7494 | 0.7711 | <0.001 | 0.4705 | 0.4628 | 0.4783 | <0.001 | 0.6156 | 0.6049 | 0.6265 | <0.001 | 0.6788 | 0.6666 | 0.6913 | <0.001 |
| Female (ref) | -- | -- | -- | -- | -- | -- | -- | -- | -- | -- | -- | -- | -- | -- | -- | -- |
| Male | 0.9715 | 0.9619 | 0.9813 | <0.001 | 0.9487 | 0.9393 | 0.9583 | <0.001 | 0.9614 | 0.9516 | 0.9713 | <0.001 | 1.0369 | 1.0259 | 1.0481 | <0.001 |
| White (ref) | -- | -- | -- | -- | -- | -- | -- | -- | -- | -- | -- | -- | -- | -- | -- | -- |
| Not known/missing | 0.1461 | 0.1432 | 0.1492 | <0.001 | 0.1583 | 0.1551 | 0.1616 | <0.001 | 0.1531 | 0.1500 | 0.1563 | <0.001 | 0.2580 | 0.2525 | 0.2636 | <0.001 |
| Other | 1.0244 | 0.9888 | 1.0612 | 0.18 | 1.0565 | 1.0198 | 1.0945 | 0.002 | 1.0738 | 1.0364 | 1.1125 | <0.001 | 1.1285 | 1.0876 | 1.1709 | <0.001 |
| IMD (numeric) DECILES | 0.9634 | 0.9611 | 0.9658 | <0.001 | 0.9714 | 0.9690 | 0.9738 | <0.001 | 0.9715 | 0.9691 | 0.9739 | <0.001 | 0.9730 | 0.9705 | 0.9755 | <0.001 |
| Distance (10km) | 0.8479 | 0.8239 | 0.8726 | <0.001 | 0.8578 | 0.8336 | 0.8829 | <0.001 | 0.8560 | 0.8318 | 0.8810 | <0.001 | 0.8107 | 0.7864 | 0.8357 | <0.001 |
| Distance square (100km^2^) | 1.0241 | 1.0179 | 1.0303 | <0.001 | 1.0234 | 1.0173 | 1.0296 | <0.001 | 1.0236 | 1.0175 | 1.0298 | <0.001 | 1.0277 | 1.0211 | 1.0343 | <0.001 |
| Number of long term conditions (DTC) |  |  |  |  | 1.1582 | 1.1557 | 1.1607 | <0.001 | -- | -- | -- | -- | -- | -- | -- | -- |
| Respiratory |  |  |  |  |  |  |  |  | 1.2449 | 1.2316 | 1.2585 | <0.001 | 1.1776 | 1.1643 | 1.1910 | <0.001 |
| Pre-diabetes |  |  |  |  |  |  |  |  | 1.1039 | 1.0875 | 1.1205 | <0.001 | 0.9186 | 0.9047 | 0.9327 | <0.001 |
| Diabetes |  |  |  |  |  |  |  |  | 1.1867 | 1.1698 | 1.2038 | <0.001 | 1.0491 | 1.0338 | 1.0647 | <0.001 |
| Heart disease |  |  |  |  |  |  |  |  | 1.3732 | 1.3531 | 1.3937 | <0.001 | 1.4337 | 1.4123 | 1.4555 | <0.001 |
| Atrial fibrillation |  |  |  |  |  |  |  |  | 1.4745 | 1.4495 | 1.4999 | <0.001 | 1.3931 | 1.3688 | 1.4178 | <0.001 |
| Kidney disease |  |  |  |  |  |  |  |  | 1.1394 | 1.1201 | 1.1590 | <0.001 | 1.1681 | 1.1480 | 1.1886 | <0.001 |
| Depression |  |  |  |  |  |  |  |  | 1.3953 | 1.3802 | 1.4107 | <0.001 | 1.2891 | 1.2744 | 1.3039 | <0.001 |
| Hypertension |  |  |  |  |  |  |  |  | 1.0554 | 1.0423 | 1.0687 | <0.001 | 0.9994 | 0.9867 | 1.0122 | 0.92 |
| Stroke |  |  |  |  |  |  |  |  | 1.3705 | 1.3379 | 1.4040 | <0.001 | 1.3998 | 1.3658 | 1.4348 | <0.001 |
| Number of primary care appointments |  |  |  |  |  |  |  |  |  |  |  |  | 1.0284 | 1.0281 | 1.0287 | <0.001 |
|  | Variance | SD | No. GPs |  | Variance | SD | No. GPs |  | Variance | SD | No. GPs |  | Variance | SD | No. GPs |  |
| Effect of GP surgery (random effect) | 0.03778 | 0.1944 | 91 |  | 0.04222 | 0.2055 | 91 |  | 0.0403 | 0.2007 | 91 |  | 0.1125 | 0.3354 | 91 |  |

IRR Incidence rate ratio, CI confidence interval, IMD Index of Multiple Deprivation. GP general practice
